# Formulation of a Commercial Biosurfactant for Application as a Dispersant of Petroleum and By-Products Spilled in Oceans

**DOI:** 10.3389/fmicb.2016.01646

**Published:** 2016-10-18

**Authors:** Bruno G. Freitas, Juliana G. M. Brito, Pedro P. F. Brasileiro, Raquel D. Rufino, Juliana M. Luna, Valdemir A. Santos, Leonie A. Sarubbo

**Affiliations:** ^1^Center of Sciences and Technology, Catholic University of PernambucoRecife, Brazil; ^2^Advanced Institute of Technology and InnovationRecife, Brazil

**Keywords:** biosurfactant, dispersant, emulsifying agents, stability, petroleum remediation, environmental contamination

## Abstract

Oil spills in oceans cause irreparable damage to marine life and harm the coastal populations of affected areas. It is therefore fundamental to develop treatment strategies for such spills. Currently, chemical dispersants have been used during oil spills, although these agents have been increasingly restricted due to their toxic potential. Thus, the aim of the present study was to formulate a biodegradable commercial biosurfactant for application as a dispersant. Biosurfactants are scientifically known biomolecules produced by microorganisms capable of allowing water-oil interaction. Thus, a biosurfactant was produced by the yeast *Candida bombicola* URM 3718 cultivated in industrial waste and formulated with the addition of a potassium sorbate preservative for fractionated sterilization (tyndallization) and the combination of fluent vaporization with the preservative. After formulation, samples were stored for 120 days, followed by surface tension, emulsification and oil dispersant tests in sea water. The results were promising for the biosurfactant formulated with the preservative, which demonstrated stability and an absence of toxicity in experiments with a marine indicator. The commercial biosurfactant was tested at different pH values, temperatures and in the presence of salt, demonstrating potential industrial application at a cost compatible with the environmental field. The formulation process developed in this research was patented in the Brazilian National Intellectual Property Institute (patent number BR1020140179631).

## Introduction

It is estimated that approximately 0.8 ± 0.4% of the entire world production of petroleum eventually reaches the oceans. Small spills from transport ships correspond to 98% of all losses of oil and oil by-product. Accidental spills account for the remaining 2%, which contribute to the release of approximately 400,000 tons of oil per year into the environment (Hazra et al., 2012).

As a hydrophobic material, petroleum has negative effects on the structural and functional properties of cell membranes in living organisms. When in contact with water, oil and its by-products form a thin layer on the surface that impedes the exchange of gasses between the air and water, thereby hindering the passage of sunlight and the processes of respiration and photosynthesis. Thus, the impact of hydrocarbon residue causes a fundamental collapse in the food chain. The potential threat of hydrocarbons to human health is linked to the physical and chemical properties of these compounds, which are absorbed through the skin and rapidly spread throughout the organism when ingested or inhaled (Silva et al., 2014).

Despite efforts in the creation of laws over the decades, when one speaks of petroleum and sustainable development, maritime transport with oil cargo will continue to exist, along with the potential risk of accidents involving oil and by-product spills (Barrow and Rothschild, 2002). Thus, the possibility of environmental contamination is real and imminent, with an urgent need for the development of novel technologies that can contain possible contaminations (Silva et al., 2014). Contamination by petroleum and its by-products can be treated through physical, chemical, or biological methods. However, new guidelines for the recovery of water and soil have restricted the use of chemical products (Muthusamy et al., 2008). Thus, bioremediation has gained ground in recent years (Marchant and Banat, 2012).

Bioremediation played an important role in the cleaning of the spillage of 41 million liters of oil by the oil tanker Exxon Valdez in the Gulf of Alaska in 1989, giving rise to the development of this technology and demonstrating that there are good reasons to believe in the effective application of this method for the treatment of future oil spills under appropriate circumstances. While it was difficult to evaluate the effects of treatment due to the heterogeneity of the contamination, other studies have demonstrated the importance of the use of surfactants to enhance the biodegradation of oil (Silva et al., 2014). Thus, surfactant compounds have become an attractive alternative for the removal of hydrophobic contaminants generated by the petroleum industry (Santos et al., 2014; Chaprão et al., 2015).

Surfactants or detergents, as they are more commonly known, are amphipathic compounds that partition preferentially at the interface between fluid phases with different degrees of polarity, which is of use in different industrial applications (Geys et al., 2014). Surfactants have a molecular structure with hydrophilic and hydrophobic groups that exhibit properties, such as adsorption, the formation of micelles, the formation of emulsions, foaming action, solubility, and detergency. These properties lead to a reduction in the surface tension of water, thereby allowing greater interaction with the oily phase and, consequently, greater access of microorganisms to emulsified droplets as well as acting on the containment of oil through dispersant and/or aggregating properties (Campos et al., 2013).

Most commercially available surfactants are synthesized from petroleum by-products. However, the need for environmental protection and environmental control legislation has motivated the development of natural compounds as an alternative to existing products. Thus, surfactants of miciobiological origin produced mainly by bacteria and yeasts has piqued the interest of researchers and are known as biosurfactants (Mao et al., 2015). Biosurfactants comprise a large variety of chemical structures, such as glycolipids, lipopeptides, protein-polysaccharide complexes, phospholipids, and fatty acids produced by microorganisms grown on insoluble (oils, residues, and hydrocarbons) and soluble (carbohydrates) substrates (Fracchia et al., 2012).

The first studies involving biosurfactants began in the 1980s. Since then, research has led to the development and commercialisation of several products, such as Surfactin, which is a lipoprotein produced by the bacterium *Bacillus subtilis*, and rhamnolipids, which are a group of glycolipids produced by the bacterium *Pseudomonas aeruginosa* and sold by the Jeneil Biosurfactant Company (USA). Although extremely efficient, these two biosurfactants are sold at a high cost due to the substrates employed for their production and the purity level required for application in the fields of pharmaceutics and medicine (Santos et al., 2016).

One of the options for reducing the costs related to biosurfactant production is to replace commonly used substrates with low-cost raw materials, such as industrial waste. Thus, the use of residue from oil lubricants, waste fry oil, starch residue, waste products from the dairy industry, sugarcane molasses, etc., has been proposed for the production of new biosurfactants with potential industrial application (Sarubbo et al., 2015). The availability and type of raw material can contribute considerably to the production cost. It has been estimated that raw materials account for 10–30% of the overall cost of a biotechnological product. With regard to purification, which accounts for 60% of the overall production cost, such processes are not computed when biosurfactants are applied to the environment in crude form, which eliminates these expensive steps (Santos et al., 2016).

In recent years, studies directed at the production of biosurfactants have intensified due to the characteristics of these compounds, such as biodegradability, low toxicity, specificity and stability under extreme conditions of temperature, pH and salinity. Thus, the petroleum and petrochemical industries stand out as the major fields of application for biosurfactants. The removal of residue and fractions of heavy oils requires washing with solvents or manual washing, both of which are dangerous, slow and expensive, as the residue and fractions of heavy oils that drift to the bottom of oil tanks are highly viscous and cannot be removed through conventional pumping. The use of biosurfactants is an alternative to this cleaning process, as these compounds reduce viscosity through the formulation of oil/water emulsions, thereby facilitating the pumping of residues and the recovery of crude oil after breaking down the emulsion (Hu et al., 2013). Thus, the cleaning of soil and water contaminated by oil spills, the removal of oily residue from storage tanks and the general increase in oil recovery processes from storage tanks constitute possible applications for biosurfactants. These natural compounds could also be used as corrosion inhibition agents for equipment, oil pipes and the tanks of transport trucks (Muthusamy et al., 2008).

It is therefore of fundamental importance to develop strategies that allow the production and consequent application of biosurfactants on an industrial scale. Thus, the aim of the present study was to formulate a commercial biosurfactant produced by the yeast *Candida bombicola* cultivated in industrial waste as substrate with the aim of applying this biomolecule as a commercial dispersant to assist in remediation processes of hydrophobic pollutants by industries that invest in the development of a “bioremediator” with application for oil spills in oceans.

## Materials and Methods

### Microorganism

*Candida bombicola* URM 3718 was obtained from the culture collection of the Mycology Department of the Federal University of Pernambuco, Brazil. The microorganism was maintained at 5°C on yeast mold agar slants containing (w/v) yeast extract (0.3%), malt extract (0.3%), tryptone (0.5%), D-glucose (1.0%), and agar (5.0%). Transfers were made to fresh agar slants each month to maintain viability.

### Substrates

Three types of industrial waste were used as substrates to produce the biosurfactant. Sugarcane molasses was obtained from the São José plant in the municipality of Igarassu (state of Pernambuco, Brasil). Corn steep liquor was acquired from Corn Products do Brasil (municipality of Cabo de Santo state of Pernambuco, Brasil) and soy waste frying oil was obtained from a local restaurant.

### Growth Conditions

The biosurfactant production conditions used in this work were previously established according to Luna et al. (2016). The inoculum of *C. bombicola* was prepared by transferring cells grown on a slant with 50 mL of yeast mold broth (YMB). The seed culture was incubated for 24 h at 28°C and agitated at 200 rpm. The yeast was cultivated in a submerged culture with shaking in a MA 832 shaker (Marconi LTDA., Brazil). The basal medium was composed of 5% sugarcane molasses, 5% waste frying oil, and 3% corn steep liquor dissolved in distilled water. The medium was sterilized by autoclaving at 121°C for 20 min. The final pH of the medium was 6.0 and the surface tension before inoculation was 50 mN/m. The inoculum (5%, v/v) was added to the cool medium at the amount of 10^4^ cells/mL. Cultivation was carried out in Erlenmeyer flasks at 30°C with shaking at 180 rpm for 120 h. Samples were withdrawn for analyses at regular intervals. All assays were carried out in triplicate and did not vary more than 5%.

### Formulation of Biosurfactant

After fermentation, the broth was centrifuged at 5 000 rpm for 20 min for the removal of the cells. The cell-free broth with crude biosurfactant (5 g/L) was submitted to different conservation methods: (a) addition of 0.2% potassium sorbate; (b) heating to 80°C for 30 min (fluent vapor), followed by the addition of 0.2% potassium sorbate; and (c) sterilization at 121°C for 30 min over three consecutive days (fractionated tyndallization). After the treatment of the crude biosurfactant in accordance with the conservation methods, the broth was stored at room temperature (28–30°C) for 120 days, with samples withdrawn at 15, 30, 45, 90, and 120 days to determine stability. Tests for the determination of surface tension, emulsification activity and the dispersant capacity of motor oil in ocean water were performed to select the best conservation method.

### Surface Tension Measurements

Changes in surface tension were monitored in the cell-free broth by the ring method using a Sigma 700 Tensiometer (KSV Instruments LTD – Finland) at controlled room temperature (27°C). The instrument was calibrated against Mill-Q-4 ultrapure distilled water (Millipore, Kankakee, IL, USA). Prior to use, the platinum plate and all the glassware were sequentially washed with chromic acid, deionised water and acetone and flamed with a Bunsen burner.

### Emulsification Activity with Motor Oil

The emulsification index was measured using the method described by Cooper and Goldenberg (1987), whereby 2 mL of motor oil obtained from a local automotive manufacturer in the city of Recife, Brazil, was added to 2 mL of the cell-free broth in a graduated screwcap test tube and vortexed at high speed for 2 min. Emulsion stability was determined after 24 h and the emulsification index was calculated by dividing the measured height of the emulsion layer by the total height of the mixture and multiplying by 100. Motor oil was used as contaminant oil that is commercially available for use in flex engines (gasoline, VNG, and alcohol), type SAE 20W-50, with synthetic guard (PETROBRAS, Brazil). It consists of a paraffinic base lubricating oil (a complex mixture of hydrocarbons) and performance enhancing additives. The viscosity of the oil is 98.0 cSt (at 40°C) and its density is 0.9420 g/mL (at 20°C).

### Oil Displacement Test (Dispersant Test)

The oil displacement test was carried out by slowly dropping 20 μL of motor oil onto the surface of 40 mL of sea water in a Petri dish (15 cm in diameter) until covering the entire surface area of the water. This was followed by the addition of 10 μL of the cell-free broth (crude biosurfactant) onto the surface of the oil layer. The mean diameter of the clear zones of triplicate experiments was measured and calculated as the rate of the Petri dish diameter (Ohno et al., 1993).

### Effect of Environmental Factors on the Formulated Biosurfactant Activity

After selecting the best conservation methodology, the effects of addition of different concentrations of NaCl (3 and 5%), different temperatures (40 and 50°C) for 60 min and different pH values evaluated after adjustment of the broth pH to 5 and 9 with 6.0 M NaOH or HCl on surface tension, dispersion, and emulsification were evaluated at 0, 15, 30, 45, 90, and 120 days.

### *Artemia* Assay

The toxicity assay was performed with the formulated cell-free broth (biosurfactant formulated with 0.2% potassium sorbate) using brine shrimp (the microcrustacean *A. salina*) as the bioindicator. Brine shrimp eggs were obtained from a local store and larvae were used within 1 day of hatching. The assays were conducted in 10-mL penicillin tubes containing 10 brine shrimp larvae in 5 mL of saline water (33 mg/L) per tube. The brine shrimp larvae in each tube were tested using 5 mL per concentration level of biosurfactant solution and observed for 24 h for the calculation of the mortality rate (Meyer et al., 1982). The toxicity threshold concentration (expressed as biosurfactant concentration per 100 mL of saline water) was defined as the lowest concentration to kill all tested brine shrimp. After 24 h of incubation, the surviving organisms were quantified and the 50% lethal concentration (LC50) of the samples was determined. Each test was run in triplicate and saline water was used as the control.

### Statistical Analysis

Surface tension, stability, and emulsification were determined at least three times. Mean and standard error values were calculated using the Microsoft Office Excel 2003 (Version 7). To locate significant differences, Tukey’s test (P∖0.05) was used by aid of software Statistica^®^ Version 12.0.

## Results

To offer a commercial biosurfactant, it was necessary to submit the produced biosurfactant (5 g/L) to conservation methods to evaluate its properties (surface tension and dispersion, which permit the breakdown of the oil spill and emulsification, allowing mixture in the form of droplets to facilitate the biodegradation by microorganisms in the seawater) over a period of 120 days, thereby estimating the validity of the product to be offered in a commercial form. Thus, heating methods were used separately and combined with the addition of potassium sorbate, which is a preservative that inhibits the growth of mold that is widely used in the production and conservation of foods and was tested at the same concentration used in food products.

### Evaluation of the Stability of the Biosurfactant Formulated Following Conservation Methods

**FIGURE 1:**
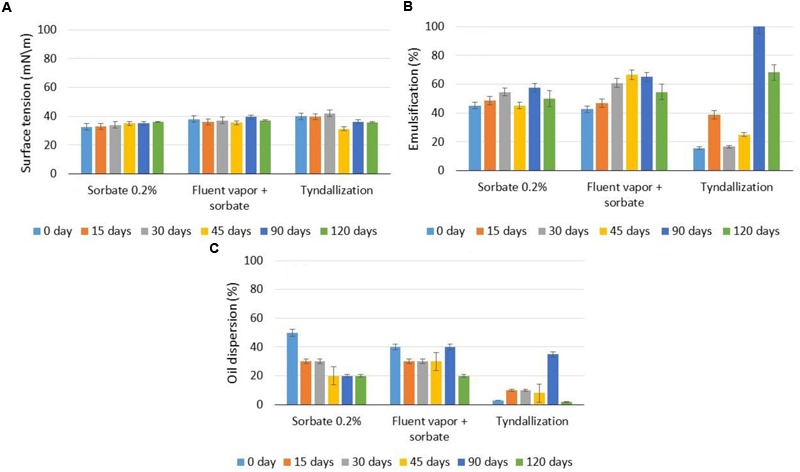
**Surface tensions **(A)**, motor oil emulsification capacity **(B)**, and motor oil dispersant capacity **(C)** of biosurfactant over 120 days of conservation with the addition of 0.2% potassium sorbate, fluent vaporization with the addition of 0.2% potassium sorbate and fractionated tyndallization at 27°C and pH 7.0.** Error bars illustrate experimental errors (standard deviations), calculated from three independent experiments.

**Figure 1** illustrates the stability of the biosurfactant in terms of the conservation methods tested under environmental conditions with regard to the reduction in surface tension, motor oil emulsification capacity and dispersant capacity of motor oil in seawater. The biosurfactant demonstrated little variation in surface tension when submitted to the three methods over 120 days of analysis, especially in the presence of potassium sorbate and sorbate plus fluent vapor (**Figure 1A**), maintaining the surface tension around 35 mN/m. The tests for the determination of the emulsification index using motor oil as the substrate for the hydrophobic moiety (**Figure 1B**) demonstrated that the crude biosurfactant emulsified 100% of the oil when submitted to tyndallization after 90 days. However, considering the importance of the maintenance of the properties throughout the storage time, the addition of potassium sorbate and fluent vaporization were the most appropriate methods for the maintenance of the emulsification capacity, which ranged between 40 and 60% over the 120 days.

The dispersant capacity of a biosurfactant is of extreme importance when the intention is to treat marine environments contaminated with hydrocarbons, as this property helps accelerate the natural dispersion and degradation of the oil spill by breaking down the droplets, consequentially promoting a larger surface area for all degradation processes or photo-oxidation (Hazra et al., 2012). As seen in **Figure 1C**, fluent vaporization allowed dispersion between 30 and 40% over 90 days, whereas the addition of potassium sorbate allowed dispersion between 30 and 50% for the first 30 days of storage. Although the surface tension of the biosurfactant was maintained stable after tyndallization, the emulsification ability and the dispersion capacity changed along time and did not show an expected behavior. Interesting results were observed only after 90 days and this is not interesting under the commercial point of view.

From the results shown in **Figure 1**, the conservation method with the addition of potassium sorbate was selected for the formulation of the commercial biosurfactant for application in oil spills at a tropical temperature (28–30°C) in a medium with a pH around neutrality, as expected for seawater.

### Evaluation of the Stability of the Biosurfactant Formulated with the Selected Preservative

**FIGURE 2:**
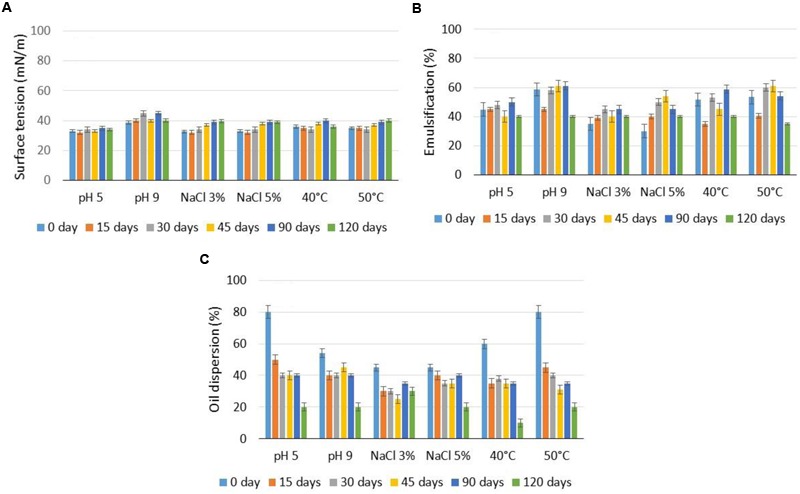
**Surface tensions **(A)**, motor oil emulsification capacity **(B)**, and motor oil dispersant capacity **(C)** of biosurfactant formulated with 0.2% potassium sorbate over 120 days under the influence of pH, temperature and the addition of NaCl.** Error bars illustrate experimental errors (standard deviations), calculated from three independent experiments.

For the application of the biosurfactant formulated with potassium sorbate under extreme conditions, the commercial product was analyzed under different pH levels, temperatures and the addition of NaCl (**Figure 2**).

The formulated biosurfactant generally demonstrated little differentiation in surface tension under the conditions tested (35 mN/m), with the most evident variations found after 45 days of conservation when submitted to different pH, salinity and temperature conditions (**Figure 2A**). In the tests for the determination of the emulsification index using motor oil (**Figure 2B**), the formulated biosurfactant maintained emulsifying capacity under the conditions tested (40–60%), with specific variations under the different pH values, salinities and temperatures used. With regard to dispersant capacity, this property was determinant in the validity of the formulated biosurfactant for 90 days, which was the period that allowed dispersal of approximately 40%, as the biosurfactant conserved with potassium sorbate lost its dispersant capacity in an accentuated manner after this storage time (**Figure 2C**). Thus, the validity of the formulated biosurfactant should be evaluated based on the application conditions.

**FIGURE 3:**
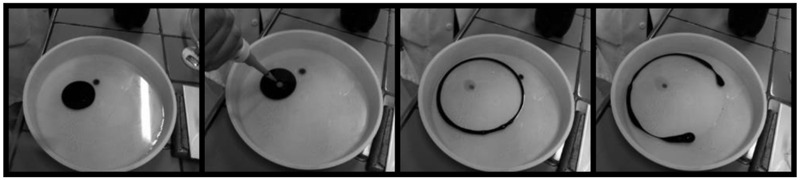
**Illustration of motor oil dispersant capacity in seawater with action of the formulated biosurfactant**.

**Figure 3** illustrates the dispersant capacity of the formulated biosurfactant.

The stability of *Candida* surfactants had been described (Rufino et al., 2008; Luna et al., 2013; Santos et al., 2013). However, most results found were obtained after short periods and thus the data obtained in this work are very difficult to be compared with the literature.

### Evaluation of Toxicity of Formulated Biosurfactant to Brine Shrimp (*A. salina*)

Companies that manufacture dispersants are required to submit information on the toxicity and percentage effectiveness of their products to the U.S. Environmental Protection Agency’s (U.S. EPA) contingent to listing their products on the National Contingency Plan (NCP) Product Schedule^1^. Short-term acute toxicity tests using consistent methodologies and test organisms provide important and fundamental information to the U.S. EPA. These tests provide an improved understanding of acute toxicological effects associated with dispersant use and helps inform future decision making.

Thus, in the present study, the acute toxicity of the biosurfactant from *C. bombicola* was examined using a brine shrimp (*A. salina*). The tests demonstrated that the biosurfactant was not toxic to this microcrustacean since the formulated cell-free broth demonstrated only 40% lethality. However, Wang et al. (2005) found an algicidal potential of rhamnolipids from *P. aeruginosa* against harmful “bloom” algae (*Heterosigma akashiwo*). The toxicity tests with the surfactant JE1058BS produced by the bacteria *Gordonia* sp. for two species of marine larvae [*Mysidopsis bahia* (shrimp) and *Menidia beryllina* (fish)] also demonstrated the low toxicity of the biosurfactant (Saeki et al., 2009).

## Discussion

The major concern regarding the use of biosurfactants in place of chemical surfactants is the production costs, which can, however, be kept low by using cheap raw materials in the fermentation medium. The yield factor is also another critical parameter which affect the economic aspects. Another essential condition for using biosurfactants is that such compounds should be produced by non-pathogenic microorganisms, as the sophorolipids, which are a group of extracellular biosurfactants produced by several non-pathogenic species of *Candida* yeast. In comparison to other biosurfactants (e.g., rhamnolipids, which are produced by pathogenic microorganisms), sophorolipids can be produced by non-pathogenic species of *Candida* and at much larger quantities (Van Bogaert et al., 2007). Moreover, these compounds can be produced with the use of low cost fermentative feedstock, such as animal fat (Deshpande and Daniels, 1995; Santos et al., 2013), deproteinized cheese whey (Daniel et al., 1998), soy molasses (Solaiman et al., 2007), restaurant waste oil (Shah et al., 2007; Batista et al., 2010), etc., by this yeast to reduce the production cost. Thus, the biosurfactant from *C. Bombicola* cultivated in a medium formulated with industrial waste with capacity to reduce the surface tension of the medium to 31 mN/m was tested as a commercial product for application in oil spills.

### Prospects for the Commercial Use of the Formulated Biosurfactant

Different technologies are employed to clean regions contaminated by petroleum and its by-products. In general, rehabilitation technologies can be grouped into physicochemical processes and biological methods. In the case of contaminated water, the pump-and-treat system is the most commonly used method and consists of the removal of the contaminated water through pumping, followed by *ex situ* treatment using conventional methods, generally adsorption to activated carbon. Although this process is efficient, it has serious limitations, mainly related to its non-destructive behavior, as this method merely leads to the generation of saturated solids with non-destroyed pollutants. Moreover, the need to combine different treatment technologies makes such processes time-consuming and expensive, mainly due to the complexity of the installations (Souza et al., 2014).

The selection criteria for the most efficient cleaning methods depend on the type and quantity of the spilled oil, the climatic conditions and the surrounding environment. During any spill, the first priority is the dispersant source. The second priority is to contain the spill with buoys to prevent greater expansion to then collect the oil from the surface of the water through barriers and skimmers, which always have limitations because such equipment cannot be used in locations with high waves, strong currents or strong winds. Barriers and skimmers require extensive human labor and operation time and are only capable of recovering 10% of the oil. This recovery percentage can even fall to 1.5–2% of the spilled volume under unfavorable marine conditions (Schramm and Klüppel, 2000).

Chemical products, known as dispersants, constitute of the practical responses to oil spills at sea. These products are used to reduce the surface tension of the oil spill, allowing the wind and waves to breakdown the microscopic droplets. The droplets are dispersed throughout the seawater in the form of floating petroleum patches that can reach the coast. The development of modern dispersants began after a spill on the coast of England in 1967. Unfortunately, dispersants based on aromatic compounds produced at the time destroyed aquatic life for a distance of miles. Dispersants basically accelerate the natural dispersion and degradation of the oil spill through the solubilisation of the oily compounds. Even after the immediate mechanical collection, dispersants can be used to minimize the contamination of birds and marine animals. Dispersants are applied from water vessels or airplanes over the area affected by the spill and can treat up to 90% of a spill at a much lower of mechanical recovery rate (Shanley, 1999; Fingas, 2002), as demonstrated in **Table 1** described by Etkin (1998a).

**Table 1:** Average cleanup cost based on analysis of oil spill cost described by Etkin (1998a; 1998b).

Cleanup strategy	Average cleanup cost per tone
Mechanical, manual recovery	$12,527
Mechanical with dispersants	$13,927
Dispersants with mechanical	$2,502
Dispersants	$2,137

The most important variables associated with the use of dispersants are the effectiveness and toxicity of the dispersant and the oil dispersed in the marine environment, although current chemical dispersants cause less ecological damage than the untreated oil spill (Schramm and Klüppel, 2000). An oil dispersant consists of a mixture of different chemical products. However, surfactants constitute the largest components, since they are responsible for the dispersion. Corexit is among the chemical dispersants currently used for oil by-product spills and was used in the accident in the Gulf of Mexico. The chemical agent passed the initial test of the Product Schedule of the NCP of the U.S. Environmental Protection Agency [EPA] (2015) after the government cut its use by half. Approximately 4,059,854 L of Corexit 9500A were applied to floating oil offshore and an additional 2,914,767 L were injected directly into the oil and gas plume at the wellhead 1544 m below the surface^2^. For the test, the EPA analyzed eight products to determine how oil dispersants would affect the marine fauna. All products had harmful effects, despite the favorable performance. The EPA continues to allow the use of Corexit, but hopes to find a more environmentally friendly alternative.

Biosurfactants are emerging as a promising alternative to chemical dispersants. According to the literature, the typical cost of a commercialized biosurfactant ranges from approximately US$ 10/mg for pure Surfactin (98% purity), used in medical research, to US$ 24/kg for formulas proposed in the early 1980s for the cleaning of oil tanks and/or advance oil recovery. The current price of sophorolipids offered by Sophoron^TM^ at “Saraya” (Japan) and “Soliance” (France), which is a type of glycolipid also produced by the yeast *C. bombicola*, is approximately US $ 2.5–6.3 kg 9 (Seydlová and Svobodová, 2008; Hazra et al., 2012).

The target market is of fundamental importance to the implantation of an industrial biosurfactant production project. For medicinal and food products, production is only viable on a small-scale. However, for environmental uses, the crude fermentation broths could be a viable solution as biosurfactants do not need to be pure and can be synthesized using a blend of inexpensive carbon sources, which would allow the creation of an economically and environmentally viable technology for bioremediation processes (Santos et al., 2016).

To determine the actual possibility of the application of the biosurfactant from *C. bombicola* formulated with potassium sorbate on an industrial level, a research of prices of previously commercialized dispersants and biosurfactants was performed to raise a preliminary cost analysis. Thus, as the price of Corexit is approximately US$ 13/L (Mcmillan, 2010), the biosurfactant from, *C. bombicola*, which is a non-purified, crude product produced from non-toxic, biodegradable industrial waste under the conditions evaluated in the present study could be a promising alterative for the treatment of spilled petroleum by-products, considering the final sales prices of the biosurfactant formulated with potassium sorbate can be estimated at US $ 0.1–0.22/L. To obtain this price, the following considerations were made: (1) a reduction of 70% in the overall production cost (costs related to energy consumption, labor, substrates, purification, and equipment maintenance), as no purification steps or high substrate costs are necessary, such as the glucose employed in the production of sophorolipids; and (2) the addition of the 0.2% potassium sorbate as the preservative (US$ 14.76/Kg). We can say that through specialized cost effective applications in the petroleum industry and investigations to establish toxicity, we can look forward to biosurfactants as the molecules of the future.

## Conclusion

The biosurfactant formulated with the preservative potassium sorbate maintains its tensioactive properties over a long storage period at a sufficient level to ensure its application as a non-toxic dispersant of petroleum and by-product spills and demonstrates commercial potential at a compatible price in environmental applications. Thus, this product could be considered promising in the use of marine environmental pollution control.

## Author Contributions

All authors contributed in this work. BF, JB, and PB carried out the experiments. VS analyzed the data. LS, RR, and JL designed the project and wrote the manuscript. LS performed manuscript editing and final improvement.

## Conflict of Interest Statement

The authors declare that the research was conducted in the absence of any commercial or financial relationships that could be construed as a potential conflict of interest.

## Footnotes

1 https://archive.epa.gov/bpspill/web/pdf/comparativetoxtest.final.6.30.10.pdf

2 www.restorethegulf.gov
